# Public Health Strategies for Western Bangladesh That Address Arsenic, Manganese, Uranium, and Other Toxic Elements in Drinking Water

**DOI:** 10.1289/ehp.11886

**Published:** 2008-10-07

**Authors:** Seth H. Frisbie, Erika J. Mitchell, Lawrence J. Mastera, Donald M. Maynard, Ahmad Zaki Yusuf, Mohammad Yusuf Siddiq, Richard Ortega, Richard K. Dunn, David S. Westerman, Thomas Bacquart, Bibudhendra Sarkar

**Affiliations:** 1 Better Life Laboratories, Inc., East Calais, Vermont, USA;; 2 Department of Chemistry and Biochemistry and; 3 Department of Geology and Environmental Science, Norwich University, Northfield, Vermont, USA;; 4 Bangladesh Association for Needy Peoples Improvement, Chorhash, Kushtia, Bangladesh;; 5 Laboratoire de Chimie Nucléaire Analytique et Bioenvironnementale, Université de Bordeaux 1, Gradignan, France;; 6 Department of Molecular Structure and Function, Research Institute of the Hospital for Sick Children and Department of Biochemistry, University of Toronto, Toronto, Ontario, Canada

**Keywords:** arsenic, Bangladesh, chronic arsenic poisoning, drinking water, manganese, uranium

## Abstract

**Background:**

More than 60,000,000 Bangladeshis are drinking water with unsafe concentrations of one or more elements.

**Objectives:**

Our aims in this study were to evaluate and improve the drinking water testing and treatment plans for western Bangladesh.

**Methods:**

We sampled groundwater from four neighborhoods in western Bangladesh to determine the distributions of arsenic, boron, barium, chromium, iron, manganese, molybdenum, nickel, lead, antimony, selenium, uranium, and zinc, and to determine pH.

**Results:**

The percentages of tube wells that had concentrations exceeding World Health Organization (WHO) health-based drinking water guidelines were 78% for Mn, 48% for U, 33% for As, 1% for Pb, 1% for Ni, and 1% for Cr. Individual tube wells often had unsafe concentrations of both Mn and As or both Mn and U. They seldom had unsafe concentrations of both As and U.

**Conclusions:**

These results suggest that the ongoing program of identifying safe drinking water supplies by testing every tube well for As only will not ensure safe concentrations of Mn, U, Pb, Ni, Cr, and possibly other elements. To maximize efficiency, drinking water testing in Bangladesh should be completed in three steps: 1) all tube wells must be sampled and tested for As; 2) if a sample meets the WHO guideline for As, then it should be retested for Mn and U; 3) if a sample meets the WHO guidelines for As, Mn, and U, then it should be retested for B, Ba, Cr, Mo, Ni, and Pb. All safe tube wells should be considered for use as public drinking water supplies.

Unsafe concentrations of arsenic have been discovered in tube well water throughout much of Bangladesh [British Geological Survey/Government of Bangladesh Department of Public Health Engineering (BGS/DPHE) 2001; Frisbie et al. 1999; U.S. Agency for International Development (USAID) 1997]. Residents who drink water contaminated with As are at risk for developing dermatologic diseases, skin cancers, and internal cancers and for adverse pregnancy outcomes and increased mortality (Ashraf et al. 2004; Frisbie et al. 2005). The Bangladeshi government, nongovernmental organizations, and the scientific community have responded by instituting widespread drinking water testing for As, as well as education programs designed to inform the populace about the dangers of drinking As-contaminated water. As a result, approximately 5,000,000 of the country’s 10,000,000 tube wells have been tested for As [UNICEF (United Nations Children’s Fund) 2007], and increasing numbers of villagers are becoming aware of the health risks associated with drinking As-contaminated water (Parvez et al. 2006).

Routine testing of drinking water for As is crucial for promoting public health in Bangladesh. However, two national-scale surveys of tube well water for other toxic elements revealed that As, manganese (Mn), uranium (U), boron (B), barium (Ba), chromium (Cr), molybdenum (Mo), nickel (Ni), and lead (Pb) are found at concentrations that exceed World Health Organization (WHO) health-based drinking water guidelines (BGS/DPHE 2001; Frisbie et al. 2002). Our 2002 study was prompted, in part, by clinical observations that certain As patients had more severe symptoms than would be expected given the levels of As in their drinking water, suggesting possible synergistic effects from other toxins, such as antimony (Sb), as well as deficient quantities of beneficial elements such as selenium (Se) and zinc (Zn).

Although there is much ongoing research about the distribution of As in the geologic materials of the region (Bhattacharya et al. 2002), the distribution of the other toxic elements commonly found in the region’s drinking water has received much less attention. An essential question for those charged with ensuring public health is whether drinking water with As concentrations that meet national or WHO criteria can be designated as safe without further testing for other toxic elements. It is crucial for public health policy to determine whether the concentrations of other commonly occurring toxic elements are correlated with the concentration of As. If they are, then the current practice of testing every tube well for As only might be sufficient to identify safe drinking water supplies. If the concentrations of these other toxicants are not positively correlated with the concentration of As, then testing every tube well for As alone will not identify safe drinking water supplies. Drinking water must be safe with respect to As and all other toxic elements.

## Materials and Methods

### Sample collection, preservation, and analyses

**Figure 1:**
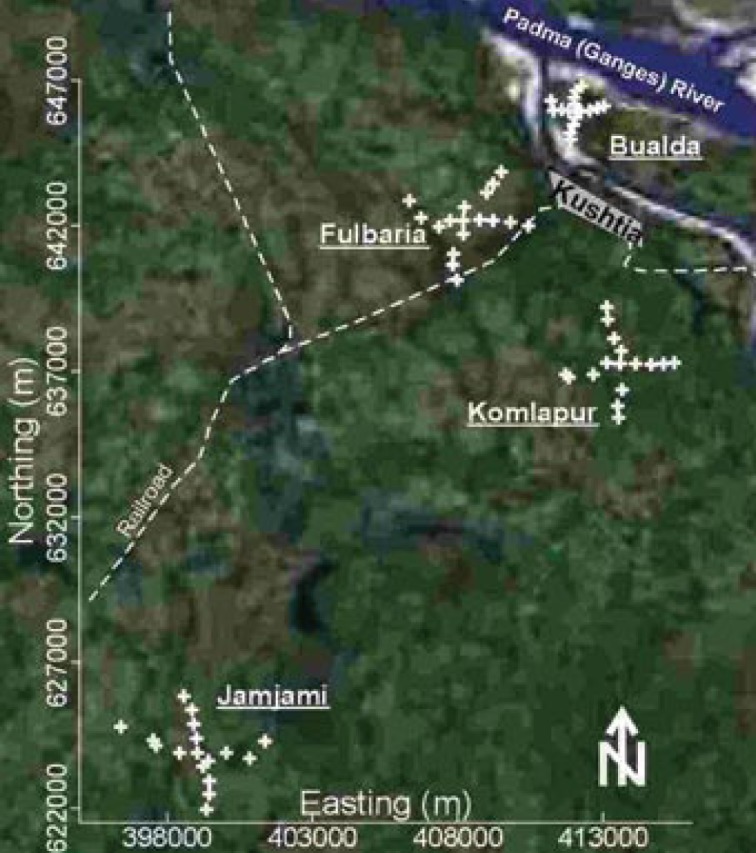
Satellite image of western Bangladesh showing the four neighborhoods where ground-water samples were collected from tube wells (GlobeXplorer, Walnut Creek, CA, USA). These four neighborhoods are centered in the villages of Bualda, Fulbaria, Jamjami, and Komlapur; each sampling location is labeled with a “+.” Kushtia is a major city.

We collected groundwater samples from four neighborhoods in western Bangladesh (Figure 1). Western Bangladesh was chosen for this study because it has some of the widest ranges of groundwater As concentrations in the country, according to our two national-scale surveys (Frisbie et al. 1999, 2002; USAID 1997). Therefore, it is a region where both drinking water testing and treatment for As are important public health strategies. We selected these neighborhoods at random within this region.

Seventy-one samples were collected from 67 randomly selected tube wells in these four neighborhoods. We collected a total of 18 samples from 17 tube wells in each of three neighborhoods (Bualda, Fulbaria, and Jamjami). We were denied access at one sampling location; therefore, 17 samples were collected from 16 tube wells in the fourth neighborhood (Komlapur). To the extent possible, the sampled tube wells in each neighborhood were distributed at 500-m intervals along perpendicular axes that radiated in four equal lengths from the center (Figure 1). Two samples were collected from the centermost tube well in each neighborhood. We averaged the results for each analyte from each of these four centermost tube wells. One sample was collected from each of the remaining tube wells. The northings and eastings of these tube wells were measured using a Global Positioning System 12 Channel Personal Navigator (Garmin International, Olathe, KS, USA).

We used established collection, preservation, and storage methodologies to ensure that each sample was representative of groundwater quality [American Public Health Association (APHA) et al. 2005; Frisbie et al. 2005]. Accordingly, all sampled tube wells were purged by pumping vigorously for 10 min immediately before sample collection. All samples were collected directly into polyethylene bottles and were not filtered. Samples were analyzed immediately after collection with pH paper, preserved by acidification to pH < 2 with 5.0 M hydrochloric acid (product no. 101256J; BDH Laboratory Supplies, Poole, UK), and stored in ice-packed coolers. The temperature of all stored samples was maintained at 0–4°C until immediately before analysis at laboratories in Dubai, France, and Vermont.

Samples were shipped to Dubai and analyzed for As by the arsenomolybdate method (Frisbie et al. 2005). The samples were then shipped to France and analyzed for Ba, Cr, Mn, Mo, Ni, Pb, Se, U, and Zn by inductively coupled plasma mass spectrometry (PlasmaQuad PQ2+ Spectrometer; Fisons/VG Analytical, Manchester, UK) (APHA et al. 2005). Finally, the samples were shipped to Vermont and analyzed for B by the azo-methine H method, iron (Fe) by flame atomic absorption spectroscopy (210VGP Atomic Absorption Spectrometer; Buck Scientific, East Norwalk, CT, USA) (APHA et al. 2005), and Sb by graphite furnace atomic absorption spectroscopy (210VGP; Atomic Absorption Spectrometer) (APHA et al. 2005).

### Interviews

The depth, age, and number of users were determined for each tube well by interviewing its owner or a principal user at the time of groundwater sampling. The interview was conducted in Bangla using a list of standard questions.

### Mapping and statistics

Contour maps were drawn by hand, using linear interpolation, to show the concentrations of As, B, Ba, Cr, Fe, Mn, Mo, Ni, Pb, Sb, Se, U, and Zn, as well as pH, depth of tube well, age of tube well, and number of users per tube well, for each of the four neighborhoods. We interpreted these maps visually to help make hypotheses about the effects of geology on the distributions of these elements in ground-water. We used standard methods of linear regression to test these hypotheses (Neter et al. 1985; Snedecor and Cochran 1982).

## Results and Discussion

### The distributions and health risks of toxic elements

All 71 groundwater samples from Bualda, Fulbaria, Jamjami, and Komlapur were analyzed for every toxic element that has ever been found to exceed WHO health-based guidelines in Bangladesh’s drinking water: As, B, Ba, Cr, Mn, Mo, Ni, Pb, and U (BGS/DPHE 2001; Frisbie et al. 2002). In this study, we found concentrations of As, Cr, Mn, Ni, Pb, and U that exceeded WHO health-based drinking water guidelines. Conversely, we found that B, Ba, and Mo levels did not exceed these guidelines. In addition, we analyzed all samples for Fe, Sb, Se, Zn, and pH (Tables 1, 2). A list of all of these elements follows, with elements arranged from the most to the least significant health risk in this study. We then summarize the toxicity of these elements and review the rationale for WHO health-based drinking water guidelines.

### Arsenic

Chronic As poisoning is the most significant health risk caused by drinking water from these four neighborhoods. Arsenic concentrations ranged from < 7 μg/L to 590 μg/L, with 33% of tube wells exceeding the 10 μg/L WHO drinking water guideline (Table 1; WHO 2004, 2006). Drinking water with 10 μg/L As has been associated with three extra deaths per 5,000 people from skin cancer (WHO 1996a, 1996b) and 10 extra deaths per 5,000 people from bladder, liver, or lung cancer (Morales et al. 2000). In addition to these cancers, chronic As poisoning has been associated with melanosis, leukomelanosis, keratosis, hyper keratosis, and nonpitting edema in Bangladesh (Frisbie et al. 2005).

### Manganese

Mn concentrations ranged from 160 μg/L to 2,400 μg/L, with 78% of tube wells exceeding the 400 μg/L WHO health-based drinking water guideline (Table 1) (WHO 2004, 2006). Mn is required for human nutrition; however, the accumulation of Mn may cause hepatic encephalopathy in humans (Layrargues et al. 1998). The chronic ingestion of Mn in drinking water is associated with neurologic damage in humans (Kondakis et al. 1989; WHO 1996a, 1996b). The WHO guideline for Mn in drinking water was calculated using the no observed adverse effects level (NOAEL) for these neurologic effects in humans and laboratory animals (WHO 1996b, 2004). As worldwide life expectancy increases, chronic neurologic diseases such as parkinsonian disorders associated with Mn exposure are likely to increase, especially in developing countries (Dorsey et al. 2007; Ferri et al. 2005; He et al. 2005). Thus, high intake of Mn by Bangladeshis may increase parkinsonian disorders associated with Mn exposure.

### Uranium

U concentrations ranged from < 0.2 μg/L to 10 μg/L, with 48% of tube wells exceeding the 2 μg/L WHO health-based drinking water guideline (Table 1). This WHO guideline was calculated using the lowest observed adverse effects level (LOAEL) for kidney lesions in male laboratory rats (WHO 1998a, 1998b). The carcinogenic effect of U in drinking water at natural isotopic abundance (^238^U at 99.2830%, ^235^U at 0.7110%, and ^234^U at 0.0054%) has not been adequately studied in humans and experimental animals (Weast et al. 1983; WHO 1998a, 1998b).

The first study on humans of the effects of chronic U ingestion from drinking water showed adverse kidney function, with the proximal tubule as the site of toxicity (Zamora et al. 1998). Later, a much larger study on exposure of humans to U in drinking water revealed nephrotoxic effects even at low concentrations without a clear threshold (Kurttio et al. 2002). In another study, the same authors found that people who drank water with elevated concentrations of U had indications that, in addition to kidneys, bone may be another target of toxicity (Kurttio et al. 2005).

### Lead

Pb concentrations ranged from < 0.2 μg/L to 17 μg/L, with 1% of tube wells exceeding the 10 μg/L WHO health-based drinking water guideline (Table 1) (WHO 2004, 2006). The WHO drinking water guideline for Pb was calculated using the lowest measurable retention of Pb in the blood and tissues of human infants (WHO 1996a, 1996b). Pb is a “possible human carcinogen” because of inconclusive evidence of human carcinogenicity and sufficient evidence of animal carcinogenicity. Oral exposure to Pb has been found to increase the incidence of renal tumors in laboratory rats, mice, and hamsters (WHO 1996a, 1996b, 2004, 2006). In addition, Pb also causes many noncarcinogenic disorders in humans, including, but not limited to, “neuro-toxicity, developmental delays, hypertension, impaired hearing acuity, impaired hemoglobin synthesis, and male reproductive impairment” [U.S. Environmental Protection Agency (EPA) 2008]. The effects of Pb on the central nervous system of fetuses, infants, children up to 6 years of age, and pregnant women can be especially serious (WHO 1996a, 1996b).

### Nickel

Ni concentrations ranged from 0.5 μg/L to 570 μg/L, with 1% of tube wells exceeding the 70 μg/L WHO health-based drinking water guideline (Table 1). This WHO guideline was calculated using the LOAEL in a study of oral exposure in fasting patients (WHO 2006). Ni compounds are “carcinogenic to humans” by inhalation exposure. In contrast, the carcinogenic effects of Ni in drinking water for humans have not been adequately studied. Ni in drinking water did not increase the incidence of tumors in laboratory rats (WHO 1998a, 1998b, 2006).

### Chromium

Total Cr concentrations ranged from < 0.5 μg/L to 100 μg/L, with 1% of tube wells exceeding the 50 μg/L WHO drinking water guideline (Table 1) (WHO 2004, 2006). The International Agency for Research on Cancer (IARC) has cate gorized Cr(VI) as “carcino genic to humans” and Cr(III) as “not classifiable” (IARC 1987); however, the U.S. EPA (1996) listed total Cr in drinking water as having “inadequate or no human and animal evidence of carcinogenicity.” The WHO has stated that the 50 μg/L drinking water guideline for total Cr is unlikely to cause significant health risks (WHO 1996a, 1996b).

### Boron

B concentrations ranged from < 50 μg/L to 440 μg/L, with no tube wells exceeding the 500 μg/L WHO health-based drinking water guideline (Table 1) (WHO 2004, 2006). However, 5.3% of Bangladesh’s tube wells exceeded this guideline in a national-scale survey (BGS/DPHE 2001).

### Barium

Ba concentrations ranged from 28 μg/L to 690 μg/L, with no tube wells exceeding the 700 μg/L WHO health-based drinking water guideline (Table 1) (WHO 2004, 2006). However, 0.3% of Bangladesh’s tube wells exceeded this guideline in a national-scale survey (BGS/DPHE 2001).

### Iron

Fe concentrations ranged from < 40 μg/L to 66,000 μg/L (Table 1). The WHO has not established a health-based drinking water guideline for Fe (WHO 2004, 2006). However, high body Fe stores and high dietary intakes of Fe are associated with hepato cellular carcinoma in humans (Marrogi et al. 2001) and mammary carcinogenesis in female Sprague-Dawley rats (Diwan et al. 1997). Bangladeshis ingest approximately 12%, 62%, and 26% of their dietary Fe from drinking water, eating rice, and ingesting soil, respectively; in Bangladesh, Fe is ingested at almost twice its recommended dietary allowance (Ortega et al. 2003).

### Molybdenum

Mo concentrations ranged from 0.5 μg/L to 7.8 μg/L, with no tube wells exceeding the 70 μg/L WHO health-based drinking water guideline (Table 1) (WHO 2004, 2006). In contrast, an unspecified percentage of Bangladesh’s tube wells exceeded this WHO guideline in a national-scale survey (BGS/DPHE 2001).

### Antimony

Sb concentrations ranged from < 0.5 μg/L to 6.2 μg/L, with no tube wells exceeding the 20 μg/L WHO health-based drinking water guideline (Table 1) (WHO 2004, 2006). However, 81% of the samples with detectable concentrations of As had detectable concentrations of Sb (Table 2). Sb in drinking water has been reported to modulate the toxicity of As (Gebel 1999). Therefore, it is possible that otherwise safe levels of Sb may magnify As toxicity.

Sb trioxide (Sb_2_O_3_) is “possibly carcinogenic to humans” by inhalation exposure. In contrast, the effect of Sb in drinking water on cancer in humans has not been adequately studied. Sb in drinking water did not increase the incidence of tumors in laboratory mice and rats (WHO 1996a, 1996b, 2004, 2006). The WHO guideline for Sb in drinking water was calculated using the NOAEL for decreased water intake, food intake, and body weight in laboratory rats (WHO 2004, 2006).

### Selenium

Se concentrations ranged from < 1 μg/L to 1 μg/L, with no tube wells exceeding the 10 μg/L WHO guideline (Table 1) (WHO 2004, 2006). Se is needed for human nutrition. Se does not appear to cause cancer, with the exception of Se sulfide, which is not found in drinking water (WHO 1996a, 1996b). The NOAEL for Se in humans is 4 μg/kg body weight per day. In this light, the WHO set the health-based guideline for Se in drinking water at 10 μg/L (WHO 2004, 2006).

Se prevents the cytotoxic effects of As (Biswas et al. 1999). Unfortunately, the food crops in Bangladesh are sometimes deficient in Se (Ortega et al. 2003), and the drinking water in Bangladesh is often deficient in Se (Frisbie et al. 2002). Therefore, it is possible that this lack of Se in food and drinking water might magnify As toxicity.

### Zinc

Zn concentrations ranged from 2.6 μg/L to 88 μg/L (Table 1). Zn is needed by all living organisms. The provisional maximum tolerable daily intake for Zn in humans is 1,000 μg/kg body weight. In this light, the WHO concluded that a health-based guideline for Zn in drinking water “is not required” (WHO 2004, 2006).

In Bangladesh, the severity of chronic As poisoning may be magnified by a lack of dietary Zn (Frisbie et al. 2002; Ortega et al. 2003). Zn promotes the repair of tissues damaged by As (Engel et al. 1994). Food, not drinking water, is the major source of dietary Zn (WHO 1996a), but the agricultural soils, food crops, and diet in Bangladesh are often deficient of Zn (Brammer 1996; Ortega et al. 2003). Therefore, it is possible that this lack of Zn in soils, food, and drinking water may magnify As toxicity.

### Ramifications for the monitoring, treatment, and distribution of drinking water

The average concentrations of toxic elements from all 67 tube wells sampled in this study are listed in Table 1. Thirty-three percent (22 of 67) of these tube wells exceed the WHO health-based drinking water guideline for As of 10 μg/L (Table 1).

### Analysis of tube wells with unsafe concentrations of As

The average concentrations of toxic elements from the 22 tube wells with unsafe concentrations of As are listed in Table 3. That is, 59%, 14%, 5%, 5%, and 5% of these 22 tube wells had unsafe concentrations of Mn, U, Pb, Ni, and Cr, respectively (Table 3). This suggests that drinking water wells with unsafe concentrations of As may also have unsafe concentrations of Mn, U, Pb, Ni, Cr, or possibly other elements.

In this neighborhood-scale study and in two national-scale studies of Bangladesh, levels of As, Mn, U, Pb, Ni, Cr, B, Ba, and Mo were above WHO health-based drinking water guidelines (Table 1) (BGS/DPHE 2001; Frisbie et al. 2002). In Bualda, increases in As concentration correlated with statistically significant increases in concentrations of Mn, Pb, Ni, Cr, and B (Table 4). In Jamjami, increases in As concentration correlated with statistically significant increases in concentrations of Pb, Ni, and Ba (Table 4). In Komlapur, increases in As concentration correlated with statistically significant increases in Cr and Ba (Table 4). Finally, in the entire region, increases in As concentration correlated with statistically significant increases in Mn, Pb, Cr, B, Ba, and Mo (Table 2).

Almost all of the home-scale drinking water treatment systems currently being used in Bangladesh have been designed to remove As but not these other toxic elements. The statistically significant increases in toxic elements in addition to As suggest that these treatment systems should be further evaluated for the removal of Mn, Pb, Ni, Cr, B, Ba, Mo, and possibly other elements.

### Analysis of tube wells with safe concentrations of As

The average concentrations of toxic elements from the 45 tube wells with safe concentrations of As are presented in Table 5. Of these 45 tube wells 87% and 64% had unsafe concentrations of Mn and U, respectively (Table 5). In fact, 93% (42 of 45) of these tube wells had unsafe concentrations of Mn, U, or both Mn and U (Table 5). This suggests that drinking water wells with safe concentrations of As may have unsafe concentrations of Mn, U, or possibly other elements. Thus, the current practice of testing every tube well only for As will not identify drinking water with safe concentrations of other toxic elements.

In response to this finding that Mn, U, and possibly other toxic elements commonly occur at unsafe concentrations even when As is at safe concentrations, we propose the following three-step testing program to provide safe drinking water in western Bangladesh, and possibly the entire country. This testing program is economical because it prioritizes the analysis of toxic elements, and analysis ends as soon as a sample is found to be unsafe for use as drinking water.

First, the toxicity and distribution of As relative to Mn, U, Pb, Ni, Cr, B, Ba, and Mo suggest that the current practice of sampling and testing every tube well in Bangladesh for As to find the safest sources of drinking water should remain the highest public health priority. Arsenic is expected to cause at least 150,000 extra cancer deaths during the life spans of the current population of Bangladesh (Frisbie et al. 2005). In contrast, the risk to public health in Bangladesh is smaller for Mn, U, Pb, Ni, Cr, B, Ba, and Mo (Frisbie et al. 2002; WHO 1996b, 1998b). Under conditions of limited resources, testing of these toxic elements must be prioritized.

Second, the high concentrations of As, Mn, and U relative to Pb, Ni, Cr, B, Ba, and Mo suggest that if a sample meets the WHO guideline for As, it should be retested for Mn and U. This will identify tube wells with safe concentrations of As, Mn, and U for additional evaluation as a potential drinking water supply in these neighborhoods without the cost or delay of testing for all nine elements. For example, one tube well in Fulbaria, one tube well in Jamjami, and one tube well in Komlapur did not exceed WHO health-based drinking water guidelines for As, Mn, and U.

Third, if a sample meets the WHO guidelines for As, Mn, and U, then it should be retested for Pb, Ni, Cr, B, Ba, and Mo. All tube wells that do not exceed WHO guidelines for these nine elements could be used as public drinking water supplies. For example, if the three tube wells that did not exceed WHO health-based drinking water guidelines for As, Mn, and U also did not exceed any other WHO health-based drinking water guidelines, they could supply safe drinking water to the residents of each neighborhood.

Testing only for As and then asking the owners of safe tube wells to share drinking water with their less fortunate neighbors has been a highly successful public health strategy in Bangladesh. More than 90% of western Bangladeshis share drinking water (Frisbie et al. 2005). The three-step testing program builds on this success by testing for all known toxic elements in Bangladesh’s drinking water, not just As.

Unfortunately, no tube wells in Bualda met WHO guidelines for all elements; therefore, drinking water treatment will likely be required in this neighborhood. However, this testing strategy will help the residents of places like Bualda choose the safest tube wells for interim use until a treatment plant can be built.

All tube wells identified as safe by this three-step process should be used as public drinking water supplies. These safe tube wells must be periodically monitored for As, Mn, U, Pb, Ni, Cr, B, Ba, and Mo. If a tube well becomes unsafe, then an alternative drinking water supply must be identified or the unsafe water must be treated.

Our earlier national-scale survey suggested that groundwater with unsafe levels of As, Mn, U, Pb, Ni, Cr, B, Ba, and Mo extends beyond Bangladesh’s borders into the four adjacent and densely populated Indian states of West Bengal, Assam, Meghalaya, and Tripura (Frisbie et al. 2002). The present neighborhood-scale survey in western Bangladesh borders the West Bengal districts of Nadia and 24-Parganas, where aquifers with similar characteristics occur (Bhattacharya et al. 2002). Thus, we urge that a similar survey be done in West Bengal to investigate possible exposure to unsafe levels of Mn, U, Pb, Ni, Cr, B, Ba, and Mo in addition to As in drinking water.

### The relationships among As, Mn, and U

**Figure 2:**
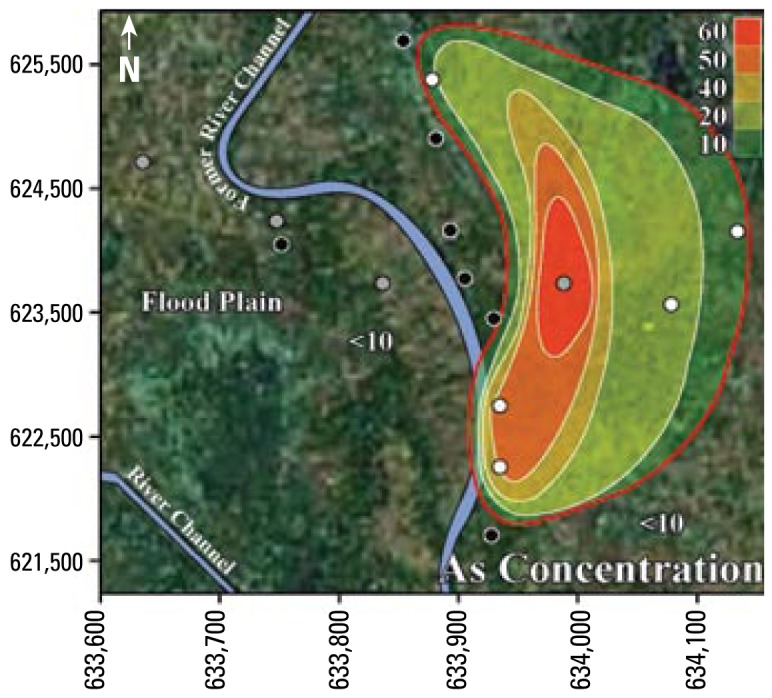
Contour map showing As concentrations (μg/L) in tube well water from Jamjami. White circles are shallow tube wells [18–27 m below ground surface (bgs)], gray circles are intermediate tube wells (28–37 m bgs), and black circles are deep tube wells (38–55 m bgs). The red contour line represents the area with wells that exceed the 10 μg/L WHO health-based drinking water guideline.

**Figure 3:**
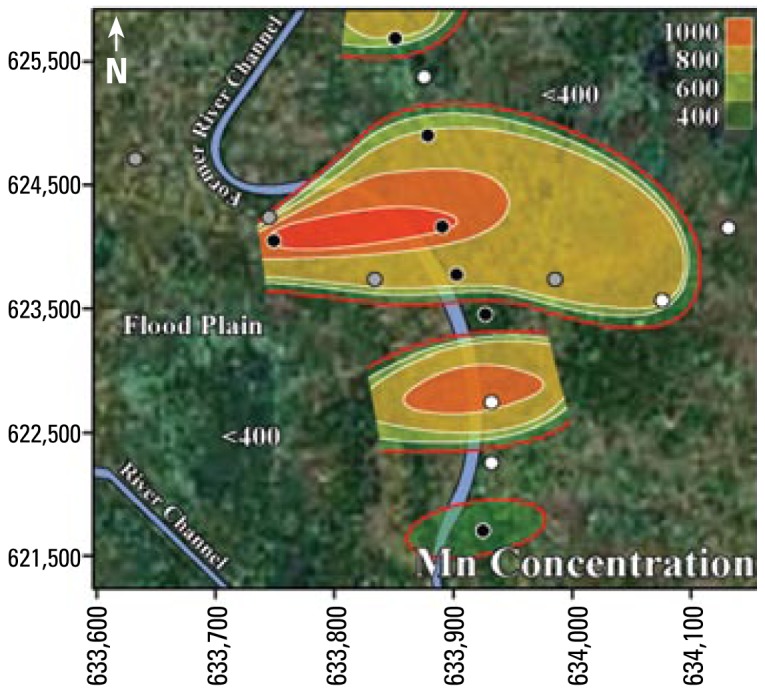
Contour map showing Mn concentration (μg/L) in tube well water from Jamjami. White circles are shallow tube wells [18–27 m below ground surface (bgs)], gray circles are intermediate tube wells (28–37 m bgs), and black circles are deep tube wells (38–55 m bgs). The red contour line represents the area with wells that exceed the 400 μg/L WHO health-based drinking water guideline.

**Figure 4:**
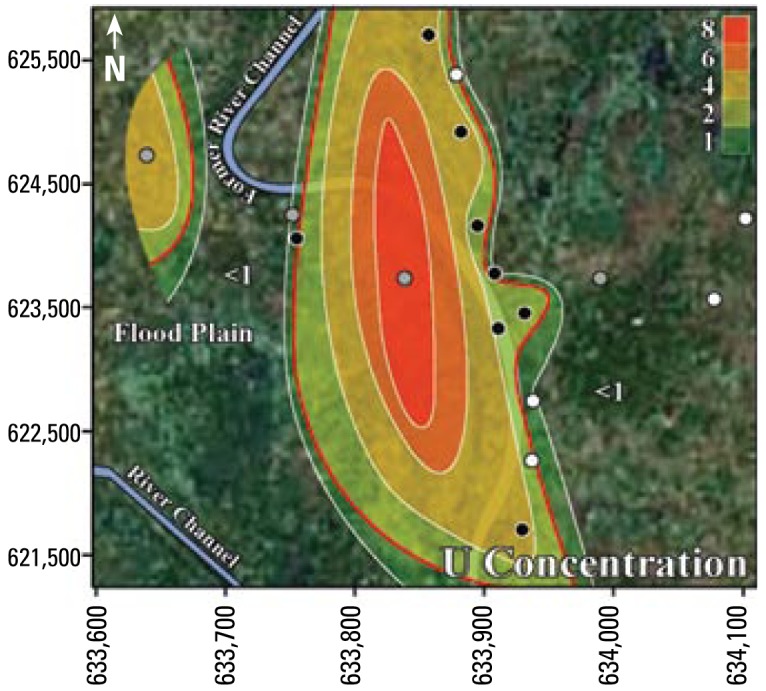
Contour map showing U concentration (μg/L) in tube well water from Jamjami. White circles are shallow tube wells (18–27 m below ground surface (bgs)], gray circles are intermediate tube wells (28–37 m bgs), and black circles are deep tube wells (38–55 m bgs). The red contour line represents the area with wells that exceed the 2 μg/L WHO health-based drinking water guideline.

The results from Tables 3 and 5 suggest that Mn is often at unsafe concentrations in Bangladesh’s tube well water. More than 50% of Bangladesh’s area has groundwater with Mn concentrations greater than the WHO health-based drinking water guideline (Frisbie et al. 2002). In addition, the contrast between 14% of tube wells with unsafe concentrations of U among the tube wells with unsafe concentrations of As (Table 3) and 64% of tube wells with unsafe concentrations of U among the tube wells with safe concentrations of As (Table 5) suggests that in western Bangladesh, drinking water with safe concentrations of U may have unsafe concentrations of As, whereas drinking water with safe concentrations of As may have unsafe concentrations of U. In summary, the drinking water in these neighborhoods generally has unsafe levels of As and Mn, or U and Mn; however, it seldom (4%, 3 of 67 tube wells) has unsafe concentrations of both As and U together. Figures 2–4 illustrate the relationships between As and Mn, U and Mn, and As and U.

The inverse trend between As and U may be caused by the variability that is characteristic of delta-alluvial plain deposits from the Bengal Delta Plain in Bangladesh and West Bengal, India. For example, in Jamjami the concentration of As decreases with depth (*p* = 0.002; Figure 2), and the concentration of U increases with depth (*p* = 0.04; Figure 4). Komlapur, to some extent, also shows these trends. In contrast, Bualda and Fulbaria show no trends between As and depth, and U and depth. The aquifers in Jamjami and possibly Komlapur contain medium- to coarse-grained sand at depth that was deposited in former river channels (Alam et al. 1990). The groundwater drawn into tube wells that are screened in these deposits may be under oxidizing conditions that remove As from ground water and release U into ground water. In contrast, the aquifers in all four neighborhoods have organic-rich mud at all depths that was deposited in flood plains (Alam et al. 1990). The groundwater drawn into tube wells that are screened in these deposits may be under reducing conditions that release As into groundwater and remove U from groundwater. Therefore, alluvial sediments of the Bengal Delta Plain make a complex three-dimensional stratigraphy of medium- to coarse-grained sand and organic-rich mud deposits that may be responsible for the inverse trend between As and U. Other factors may also be controlling release of As and U. It is important to note that in areas where drilling deeper tube wells may access water with lower levels of As, the water from these deeper tube wells may contain increased levels of U, as we found in Jamjami and Komlapur.

Despite this inverse trend, 4% (3 of 67) of the tube wells in this study had unsafe concentrations of both As and U. This is important because the home-scale drinking water filters that are being used in Bangladesh may not remove U. Also, up to 50% of Bangladesh’s tube wells exceed the WHO health-based drinking water guideline for U (BGS/DPHE 2001). The water treatment filters used in Bangladesh typically oxidize soluble As(III) to insoluble As(V) to remove As by absorption or precipitation. However, this oxidation may convert insoluble U(IV) to soluble U(VI) and potentially increase the U concentration of the water after treatment. Alternatively, this oxidation may keep dissolved U in the VI oxidation state and potentially cause no change in the U concentration of the water after treatment (Fairbridge 1972). Thus, these filters should be further evaluated for the removal of U.

## Conclusions

In this neighborhood-scale study and in two national-scale studies of drinking water tube wells in Bangladesh, concentrations of As, Mn, U, Pb, Ni, Cr, B, Ba, and Mo exceeded WHO health-based guidelines (Table 1) (BGS/DPHE 2001; Frisbie et al. 2002). In the present study, 96% of the tube wells exceeded WHO health-based guidelines for at least one of these toxic elements. The single greatest risk to public health is from As in drinking water.

Of the 67% of tube wells that had As concentrations below the WHO drinking water guideline, 87% had unsafe levels of Mn and 64% had unsafe levels of U (Table 5). Thus, testing for As alone is not sufficient to ensure safe drinking water. To address the threats to public health posed by the prevalence of multiple toxic elements, we have proposed a three-step drinking water testing program.

Of the 33% of tube wells that had As concentrations greater than the WHO drinking water guideline, 59% also had unsafe levels of Mn, 14% had unsafe levels of U, 5% had unsafe levels of Pb, 5% had unsafe levels of Ni, and 5% had unsafe levels of Cr (Table 3). Thus, water treatment systems that have been designed solely for As removal may not provide safe drinking water and should be further evaluated for the removal of Mn, U, Pb, Ni, Cr, B, Ba, and Mo.

## Figures and Tables

**Table 1:** The average concentrations of toxic elements in the groundwater of Bualda, Fulbaria, Jamjami, and Komlapur, the WHO health-based drinking water guidelines for these toxicants, and the percent of tube wells exceeding these guidelines.

Element	Average concentration (μg/L)	WHO health-based guideline (μg/L)	Percent of unsafe tube wells^a^
As	29	10	33
B	< 50	500	0
Ba	140	700	0
Cr	4.7	50	1
Fe	2,700	NA^b^	NA
Mn	800	400	78
Mo	1.4	70	0
Ni	11	70	1
Pb	0.5	10	1
Sb	1.6	20	0
Se^c^	< 1	10	0
U	2.5	2	48
Zn^c^	15	NA	NA

a Ninety-six percent (64 of 67) of these tube wells are unsafe; that is, only 4% (3 of 67) of these tube wells do not exceed any of these WHO health-based drinking water guidelines.

b The WHO has not established a health-based drinking water guideline for Fe or Zn (WHO 1996a, 1998a).

c The severity of chronic As poisoning in Bangladesh might be magnified by a lack of Se or Zn or both (Frisbie et al. 2002; Ortega et al. 2003).

**Table 2:** Correlation coefficients (*r*) for the concentrations of toxic elements in tube well water from Bualda, Fulbaria, Jamjami, and Komlapur, along with characteristics of these tube wells.

	As	B	Ba	Cr	Fe	Mn	Mo	Ni	Pb	Sb	Se	U	Zn	pH	Depth	Age	Users
As	1.00^a^																
B	0.81^a^	1.00^a^															
Ba	0.26^b^	0.40^a^	1.00^a^														
Cr	0.82^a^	0.92^a^	0.30^b^	1.00^a^													
Fe	0.82^a^	0.92^a^	0.40^a^	0.97^a^	1.00^a^												
Mn	0.46^a^	0.31^b^	0.19	0.26^b^	0.21	1.00^a^											
Mo	0.28^b^	0.05	0.16	−0.03	−0.01	0.28^b^	1.00^a^										
Ni	0.13	0.07	0.07	0.10	0.09	−0.09	−0.05	1.00^a^									
Pb	0.83^a^	0.94^a^	0.33^a^	0.98^a^	0.96^a^	0.28^b^	−0.02	0.09	1.00^a^								
Sb	0.34^a^	0.33^a^	0.56^a^	0.24	0.31^b^	0.38^a^	0.29^b^	0.40^a^	0.28^b^	1.00^a^							
Se	0.32^a^	0.31^b^	0.48^a^	0.30^b^	0.32^a^	0.31^b^	0.10	0.11	0.35^a^	0.30^b^	1.00^a^						
U	−0.02	0.07	−0.27^b^	0.04	−0.05	0.18	−0.21	−0.02	0.08	0.04	0.09	1.00^a^					
Zn	0.67^a^	0.69^a^	0.29^b^	0.73^a^	0.74^a^	0.15	0.19	0.03	0.70^a^	0.21	0.29^b^	−0.14	1.00^a^				
pH	−0.02	−0.01	−0.11	0.02	0.00	0.05	−0.10	−0.20	0.03	−0.24	0.07	0.07	0.05	1.00^a^			
Depth	−0.10	−0.07	−0.17	0.00	−0.06	0.06	−0.15	−0.22	0.01	−0.28^b^	−0.19	0.18	0.01	0.10	1.00^a^		
Age	−0.08	−0.03	−0.03	−0.05	−0.02	0.02	−0.17	−0.11	−0.03	0.01	0.16	0.27^b^	−0.17	0.11	−0.13	1.00^a^	
Users	−0.07	−0.06	−0.11	−0.05	−0.06	−0.08	−0.07	−0.03	−0.04	−0.04	0.05	0.16	−0.12	0.07	0.02	0.10	1.00^a^

Linear relationships without a footnote are not significant at either confidence level.

a Significant linear relationships at the 99% confidence level.

b Significant linear relationships at the 95% confidence level.

**Table 3:** The average concentrations of toxic elements in Bualda, Fulbaria, Jamjami, and Komlapur’s groundwater from all tube wells that exceed the WHO health-based drinking water guideline for As.

Element	Average concentration (μg/L)	WHO health-based guideline (μg/L)	Percent of unsafe tube wells^a^
As	84	10	100
B	< 50	500	0
Ba	220	700	0
Cr	9.5	50	5
Fe	7,300	NA^b^	NA
Mn	870	400	59
Mo	2.0	70	0
Ni	31	70	5
Pb	1.2	10	5
Sb	2.3	20	0
Se^c^	< 1	10	0
U	0.9	2	14
Zn^c^	21	NA	NA

a By definition, 100% (22 of 22) of these tube wells are unsafe because they all exceed the 10 μg/L WHO health-based drinking water guideline for As.

b Not applicable; the WHO has not established a health-based drinking water guideline for Fe or Zn (WHO 1996a, 1998a).

c The severity of chronic As poisoning in Bangladesh might be magnified by a lack of Se or Zn or both (Frisbie et al. 2002; Ortega et al. 2003).

**Table 4:** Correlation coefficients (*r*) for the concentration of As versus the concentrations of toxic elements in tube well water from each of the four neighborhoods in this study, along with the characteristics of these tube wells.

	As			
Element	Bualda	Fulbaria	Jamjami	Komlapur
As	1.00^a^	1.00^a^	1.00^a^	1.00^a^
B	0.91^a^	0.18	−0.03	−0.19
Ba	0.16	0.14	0.69^a^	0.74^a^
Cr	0.91^a^	0.23	0.45	0.60^b^
Fe	0.91^a^	0.21	0.61^a^	0.66^a^
Mn	0.49^b^	0.33	−0.04	−0.39
Mo	0.21	0.09	−0.24	0.27
Ni	0.91^a^	0.25	0.49^b^	0.30
Pb	0.91^a^	0.20	0.52^b^	0.24
Sb	0.37	−0.18	0.39	0.26
Se	0.40	0.14	0.47	0.53^b^
U	0.03	−0.16	−0.55^b^	−0.30
Zn	0.96^a^	−0.14	0.34	0.06
pH	0.27	−0.32	0.09	0.08
Depth	0.07	−0.09	−0.69^a^	−0.03
Age	−0.19	0.03	−0.34	0.01
Users	−0.23	−0.17	−0.33	−0.26

Linear relationships without a footnote are not significant at either confidence level.

a Significant linear relationships at the 99% confidence level.

b Significant linear relationships at the 95% confidence level

**Table 5:** The average concentrations of toxic elements in Bualda, Fulbaria, Jamjami, and Komlapur’s groundwater from all tube wells that did not exceed the WHO health-based drinking water guideline for As.

Element	Average concentration (μg/L)	WHO health-based guideline (μg/L)	Percent of unsafe tube wells^a^
As	< 7	10	0
B	< 50	500	0
Ba	110	700	0
Cr	2.4	50	0
Fe	400	NA^b^	NA
Mn	770	400	87
Mo	1.2	70	0
Ni	1.0	70	0
Pb	< 0.2	10	0
Sb	1.2	20	0
Se^c^	< 1	10	0
U	3.2	2	64
Zn^c^	12	NA	NA

a Ninety-three percent (42 of 45) of these tube wells are unsafe; that is, only 7% (3 of 45) of these tube wells do not exceed any of these WHO health-based drinking water guidelines.

b Not applicable; the WHO has not established a health-based drinking water guideline for Fe or Zn (WHO 1996a, 1998a).

c The severity of chronic As poisoning in Bangladesh might be magnified by a lack of Se or Zn or both (Frisbie et al. 2002; Ortega et al. 2003).
